# Gender identity services must better integrate members of transgender People's social networks in care

**DOI:** 10.1016/j.eclinm.2023.101829

**Published:** 2023-01-31

**Authors:** David Matthew Doyle

**Affiliations:** a Department of Medical Psychology, Center of Expertise on Gender Dysphoria, Amsterdam University Medical Centers, Location VUmc, Amsterdam, the Netherlands; b Department of Psychology, University of Exeter, Exeter, United Kingdom

Across countries, demand for gender-affirming care is rising at unprecedented rates.^1^ Services are struggling to adapt and some are failing to protect transgender health in fundamental ways. Given the complexity of this situation, both medically and politically, it is perhaps unsurprising that gender identity services have neglected to expand beyond addressing individual needs of transgender people themselves to integrate members of transgender people's social networks throughout care. Relational partners (e.g., parents, romantic partners, friends) play a key role in transgender health and, despite facing many difficulties and hurdles themselves, are not currently offered adequate support in most healthcare systems around the world (see Table 1).

**Table 1:** Factors relevant to integrating relational partners of transgender people in gender-affirming care.

Why Do Relational Partners Matter for Healthcare?	Why Might Relational Partners Struggle?	What Systemic Hurdles Stand in the Way of Integrating Relational Partners?	What Can Be Done to Better Support Relational Partners?
Social support Interpersonal factors in suicidality Familial rejection and homelessness Parental authority over transition “Spousal veto”	Transphobia Lack of knowledge/own support Courtesy stigma and blame Loss and mourning Questioning own sexual orientation/identity	Few services explicitly for relational partners Ad hoc and run with volunteers/donations Poor signposting from clinics Discriminatory public policy (e.g., reporting for “safeguarding”)	Strengthen international recommendations with best practice Develop clinical models integrating relational partners Utilize established theories Provide resources and signposting for existing services

There are a number of ways in which members of transgender people's social networks matter for outcomes of gender-affirming care, including by providing emotional and informational social support with medical, legal and personal issues.^2^ In contrast, social strain and rejection can lead to negative outcomes, such as homelessness^3^ and risk for suicide, with transgender people being approximately twice as likely to attempt suicide compared to the general population.^4^ Relational partners can also play direct roles in the care that transgender people are able to receive. In the United Kingdom, spouses of transgender people can enact what is colloquially referred to as a “spousal veto,” meaning that they can decide whether or not their partners are legally allowed to change gender. For transgender children and youth, parents exercise authority over social and medical transition, determining what steps they are permitted to take and when.

While some members of transgender people's social networks might struggle because they are overtly transphobic, even the most well-intentioned relational partners are likely to face obstacles. Relational partners report that they sometimes do not know how to respond when their loved ones are experiencing severe gender dysphoria; relational partners are expected to provide support but often they feel that there is no one supporting them in turn.^5^ For romantic partners, gender transition can lead to questioning of one's own sexual orientation and identity, which can be a transformative but difficult process,^5^ sometimes even leading to divorce and family splits.^6^ Furthermore, parents of transgender children sometimes question their own role in their child's development, facing courtesy stigma from those who “blame” them for their child's transition^6^ and in many cases, even after accepting a new gender identity, friends and family experience a sense of loss and mourning.^6^^,^^7^

In countries such as the United Kingdom and the Netherlands where services for relational partners do exist, these tend to be support groups or charities run on an ad hoc basis by volunteers that rely upon donations. Signposting to such services through formal clinics tends to be poor—in the United Kingdom, none of the gender identity clinics actively refer relational partners to such groups and only one displays publicity material in the clinic. Worryingly, public policy recently debated and enacted in places such as the United States (e.g., in Texas) threatens to investigate families of transgender children for “abuse” when they seek gender-affirming care.^8^ Such discriminatory policy not only adds to courtesy stigma faced by well-intentioned members of transgender people's social networks, it also risks enforced social disruption by dividing families and communities.

In order to better support members of transgender people's social networks, services must fully integrate relational partners throughout the care process. The World Professional Association of Transgender Health explicitly calls for involvement of relational partners in healthcare, including for child and youth assessment as well as adult sexual and mental health, in its Standards of Care.^1^ Such international recommendations should be strengthened and complemented by new guidelines for best practice. Healthcare providers should develop models of clinical pathways integrating relational partners (not only parents and romantic partners, but also wider family and friendship networks). Researchers should draw upon established theories, such as family systems theories,^9^ to test programs and clinical approaches that may aid relational partners of transgender people. Existing services, such as Depend in the United Kingdom, (H)Erkenning in the Netherlands and LISTAG in Turkey, should be provided ongoing resources and funding and be clearly signposted in their respective healthcare systems, ideally from the point of referral to gender-affirming care, rather than at entry, as waitlists currently extend for months or even years. Proper healthcare for transgender people involves attending to not only their physical and mental health, but their social health as well,^10^ necessitating integration of relational partners throughout care. Ensuring strong and supportive social relationships is a matter of health equity for transgender patients.

## Contributors

DMD is the sole author and conceptualized, wrote, reviewed and edited the commentary.

## Declaration of interests

The author declares no conflict of interests relating to the commentary.
